# Supplementary material for: Local computational methods to improve the interpretability and analysis of cryo-EM maps
Source: Nat Commun. 2021 Feb 23;12:1240. doi: 10.1038/s41467-021-21509-5 (PMC7902670; doi:10.1038/s41467-021-21509-5)
Supplement: Supplementary file 1 — Supplementary Information [file 41467_2021_21509_MOESM1_ESM.pdf]

Supplementary Information

Supplementary Note 1: Polycystin-2 (PC2) TRP channel

We compared the performance of LocSpiral with other methods, including LocalDeblur, our proposed local B-factor correction method (LocBSharpen) and the global B-factor correction approach as implemented in Relion. To compare the different results, we used metrics proposed in[1]. The results are shown in Supplementary Figure 2. In this case, we used a relatively high threshold value to visually compare the different maps. From Supplementary Figure 2, we can see that the map obtained by LocSpiral shows good connectivity and is less affected by broken or missed densities. EMRINGER[2] and cross-correlation scores (obtained using PDB 6t9n as reference) show approximately similar results for all cases, though, the highest scores are provided by LocSpiral and LocBSharpen approaches. For the sake of comparison, we also provide FSC curves calculated by comparing the different maps with the reference atomic model (PDB 6t9n). In this case, the best results at high resolutions are provided by LocalDeblur and by LocSpiral approaches.

In addition, we provide results of LocBFactor and LocOccupancy methods. In Supplementary Figure 2B, we show the obtained local B-factor map (left map) to be used for sharpening (slope of the local Guinier plot multiplied by a factor 4) and the A-map (middle map) corresponding to the local values of the logarithm of structure factors amplitudes at 15 Å. The resolution range used to estimate these maps was between 15 Å to the FSC resolution (2.96 Å). The average value inside the solvent mask of local B-factors obtained from amplitude values above the noise level (signal B-factors) gives a value of -129.76 $Å^2$, which is smaller than the value provided by Relion (-84.56 $Å^2$) computed from the unmasked reconstruction. The A-map provides

the fitted local amplitudes at 15 Å, showing the local "amount" of signal at this resolution. As expected, local B-factor map (B-map) shows that the inner parts of the protein show lower B-factors than the outer regions. Supplementary Figure 2B shows additionally the obtained local occupancy map (right map). Interesting, both the occupancy and A maps show low values in the regions occupied by detergent densities, lipid densities and cholesterol densities (please see Figure 2 in[3]), indicating the presence of compositional variability in these regions and low signal at 15 Å.

Supplementary Note 2: Immature prokaryote ribosomes

In Supplementary Figure 4 we show maps with improved contrast at high-resolution obtained after processing EMD-8441 by LocSpiral and Relion methods[4,5]. The same soft mask was applied to both maps. In the figure, we show the maps at low and high threshold values. When a low threshold value is used, it is not possible to see details in the Relion map, while at high threshold values many regions of this map are not visible. Conversely, LocSpiral approach shows high resolution features at both high and low thresholds without losing appreciable map densities.

Finally, we also show in Supplementary Figure 4 the local B-factor map (B map given by the slopes of the local Guinier plot) and the local values of the logarithm of structure factor's amplitudes at 15 Å (A map in the figure) obtained by LocBFactor approach. The average value of the local signal B-factors to be used for sharpening (slope of the local Guinier plot multiplied by 4) within a solvent mask is -394.28 $Å^2$. We obtained the B-factor estimations within a resolution range between 15 Å to the FSC resolution given by 3.7 Å. The B-factor map shows higher B-factors at the outer part of the macromolecule, corresponding to regions that are partially folded and show compositional and conformational heterogeneity and lower local resolutions, as can be seen from Supplementary Figure 4, Class 3 in[6]. As shown in the previous Spliceosome case,

regions dominated by the noise signal within the used resolution range present artefactual low B-factors (noise B-factors) which describe the noise fall off inside the resolution range. These noise B-factors appear in Supplementary Figure 4 with black colour and correspond to the lowest amplitudes (below the noise level) in the A map at 15 Å resolution.

Supplementary Note 3: B-factor analysis of low and high resolution maps

We have performed additional B-factor analysis of approximately homogeneous low- and high-resolution maps corresponding to EMD-20671 and EMD-21024. The Gold standard FSC resolution and the estimated B-factors to be used for sharpening (slope of the local Guinier plot multiplied by 4) as determined by Relion postprocessing are 16.01 Å and -97.70 Å^2 for EMD-20671, and 1.77 Å and -50.81 Å^2 for EMD-21024, respectively. Note that the B-factors for sharpening obtained by Relion are very similar for maps showing very different resolutions. Additionally, we have computed local B-factor maps from LocBFactor approach. For both maps, we used a resolution range of [15, 4] Å. The obtained averages of signal B-factors used for sharpening inside solvent masks are -1172 Å^2 and -78 Å^2 for EMD-20671 and EMD-21024, respectively. Note that the values obtained by Relion and LocBFactor for EMD-21024 are similar. Oppositely, the average B-factor obtained by LocBFactor for EMD-20671 is much lower and consistent with a map at 16.01 Å resolution than the one reported by Relion. We believe that the reason of this discrepancy is because LocBFactor filters out noise B-factors (B-factors obtained from amplitudes below the noise level for the used resolution range) while Relion does not filter regions dominated by noise within the used resolution range. Supplementary Figure 7 shows obtained B-factor maps, FSC curves and respective Guinier plots at noise and signal regions for both cases.

Supplementary Figures

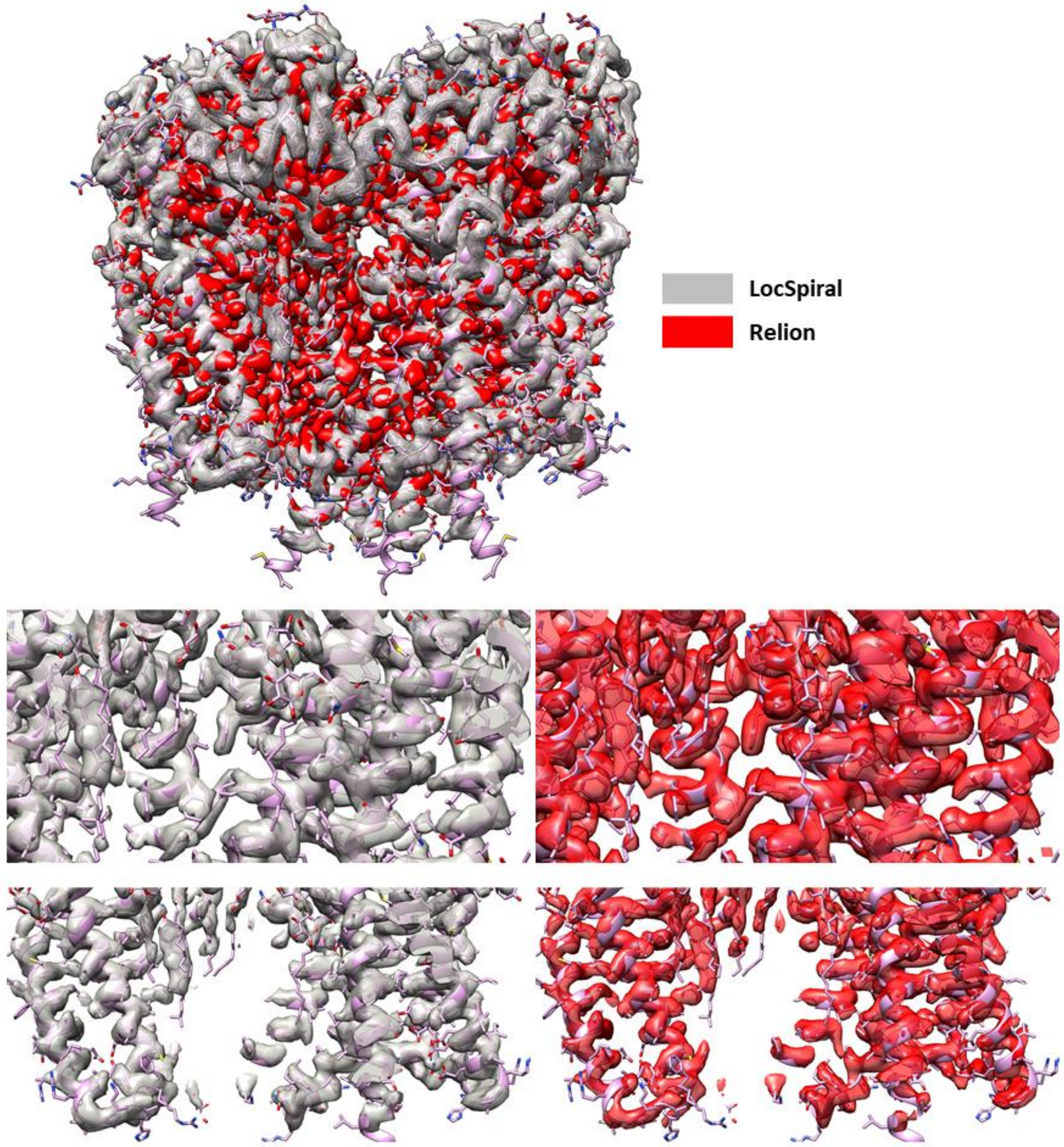

Supplementary Figure 1 Comparison between LocSpiral and Relion postprocessing maps for the TRP channel. A) Complete and overlapping LocSpiral and Relion maps shown with the corresponding atomic structure (PDB 6t9n). B) Reconstructions of corresponding regions at the core and bottom outer region of the TRP channel obtained from LocSpiral (left) and Relion (right) approaches.

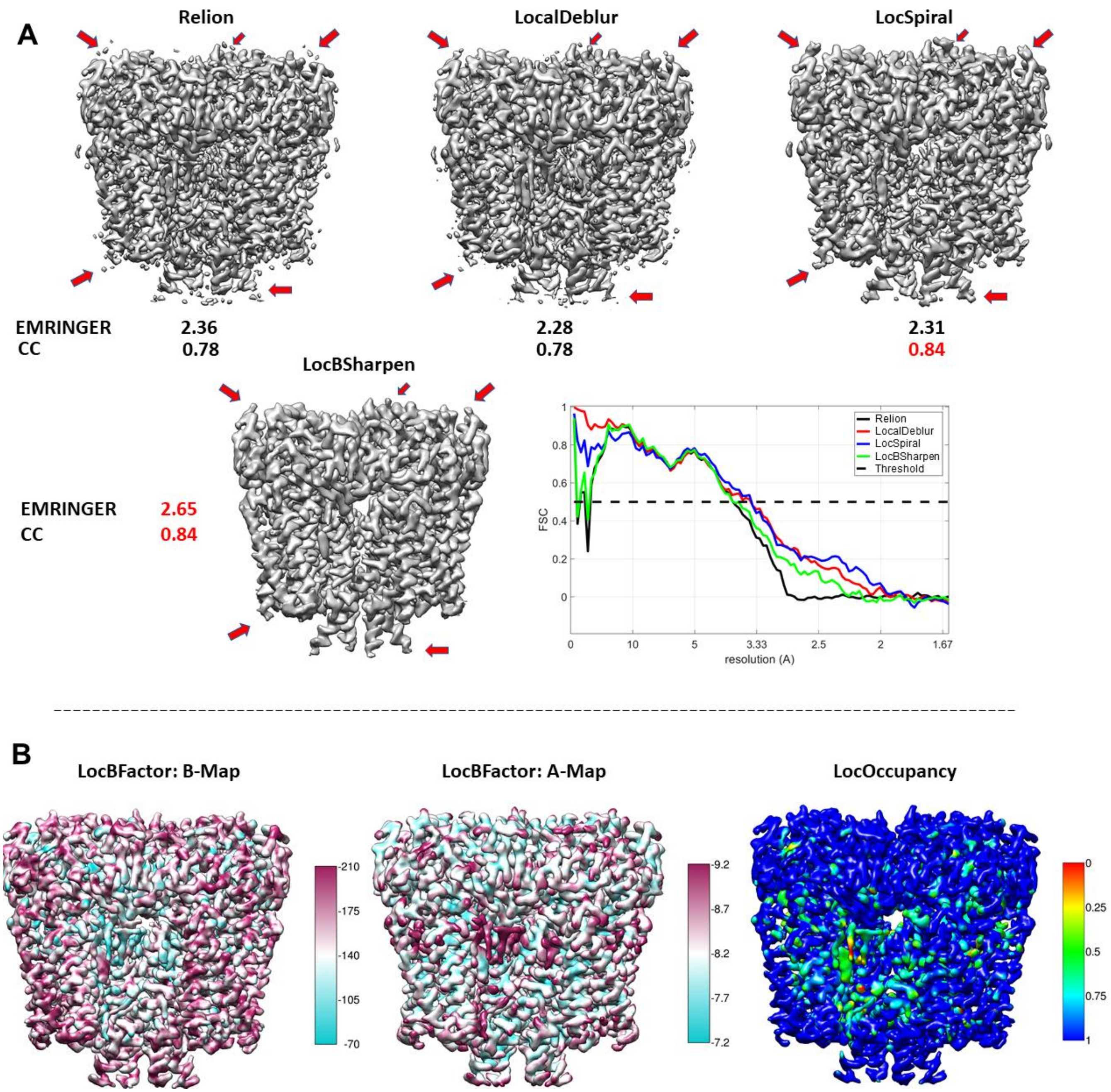

Supplementary Figure 2 Results and comparisons between different methods over the TRP channel. A) Comparison between maps obtained by different sharpening approaches: Relion, LocalDeblur, LocSpiral and LocBSharpen. Red arrows show broken or missed densities that could be seen from LocSpiral and LocBSharpen maps. Below each map, EMRINGER and cross-correlation (CC) scores calculated between obtained maps and the atomic model (PDB 6t9n) are provided. We also show FSC curves comparing the different maps with the reference atomic model (PDB 6t9n). B) Results obtained by LocBFactor (B and A maps) and LocOccupancy for the TRP channel.

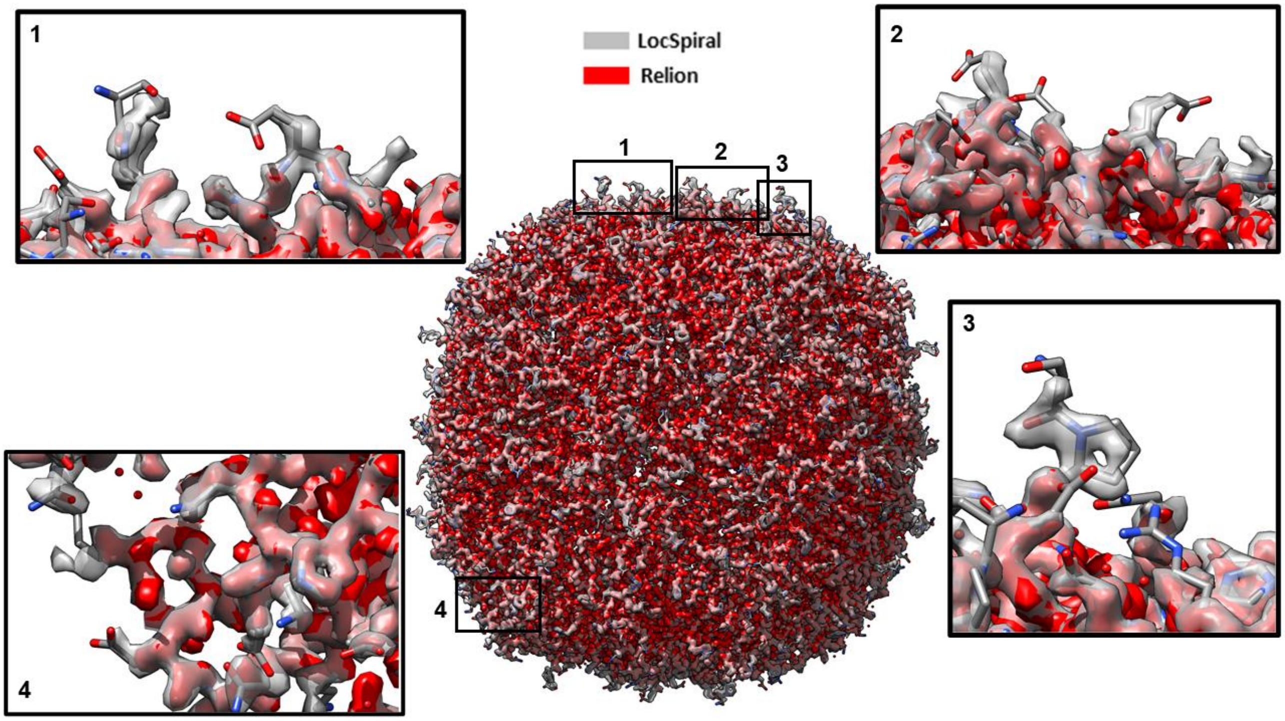

Supplementary Figure 3 Complete and superimposed sharpened maps obtained by LocSpiral (gray colour) and Relion (red colour) for EMD-9865 with the corresponding atomic structure (PDB 6v21). In the black rectangles are shown zoomed views of the regions labelled with the same index.

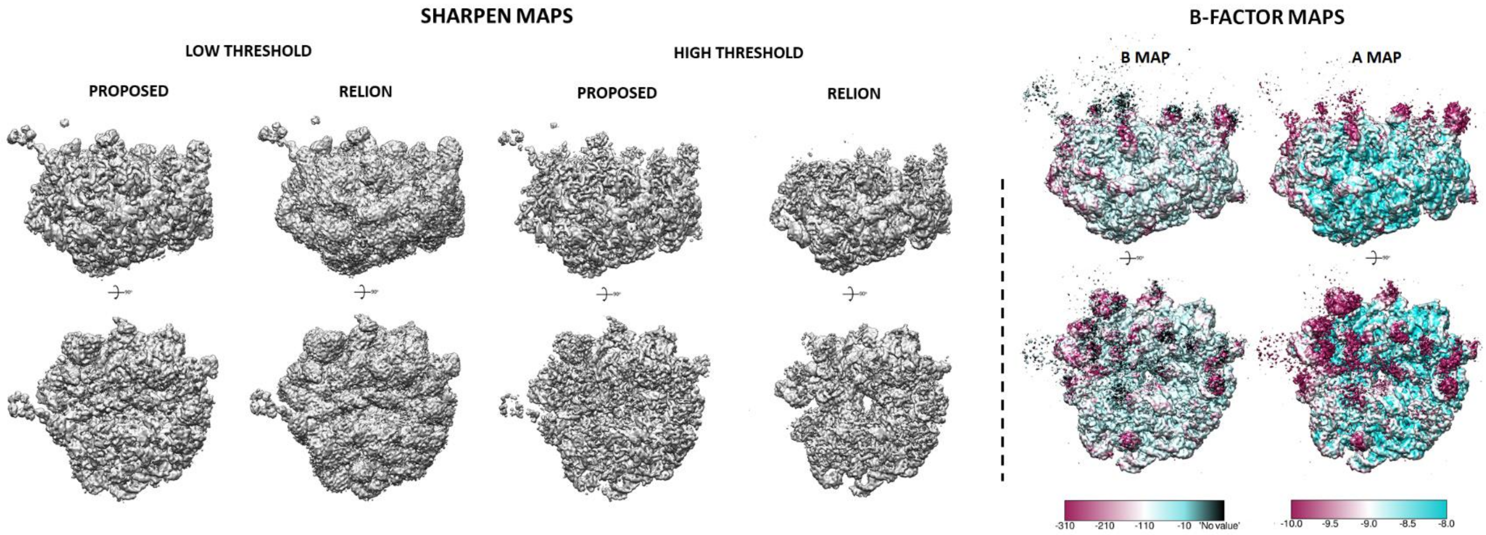

Supplementary Figure 4 Results and comparisons between different methods for the EMD-8441 immature ribosome. (Left) Obtained sharpened maps for EMD-8441 by LocSpiral and Relion. These maps are shown at low and high thresholds. (Right) Obtained B-maps (local B-factor maps corresponding to the slope of the local Guinier plots) and A-maps (local values of the logarithm of structure factor amplitudes at 15 Å) of EMD-8441 at different orientations. In these figures, noise B-factors (B-factors obtained from amplitudes below the noise level for the used resolution range) are filtered out and appear with black color.

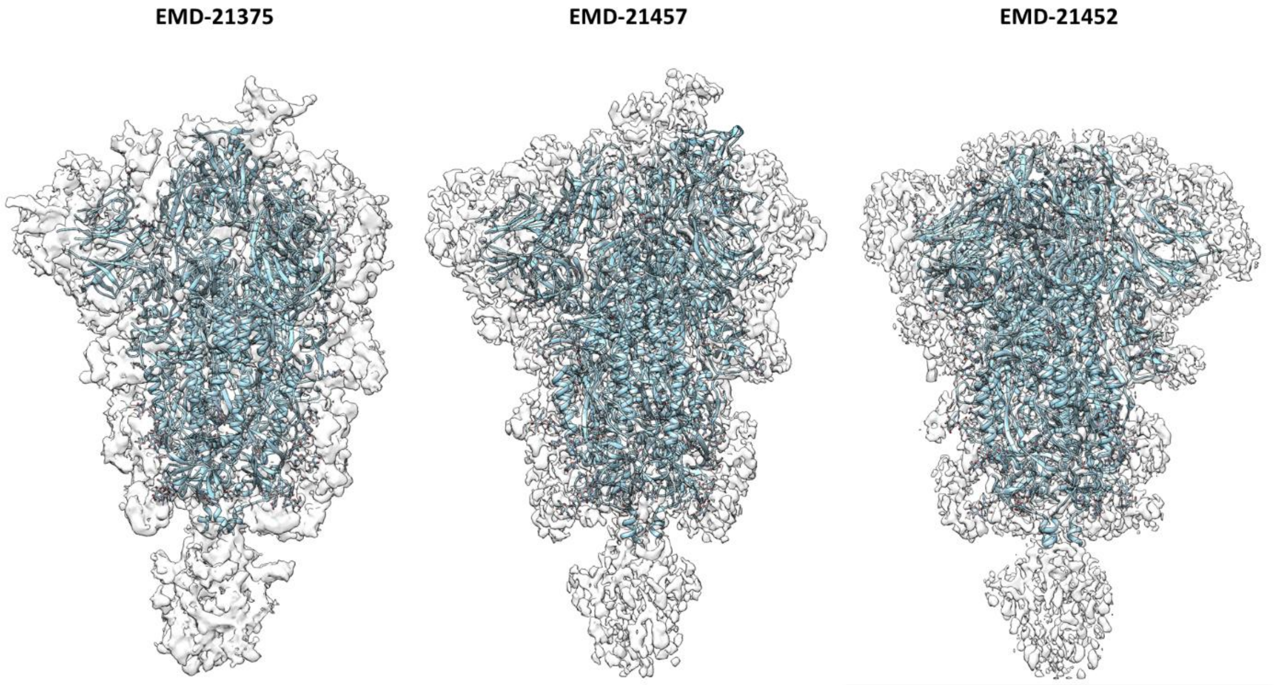

Supplementary Figure 5 Improved maps obtained by LocSpiral from EMD-21375, EMD-21457, EMD-21452 and corresponding fitted atomic models.

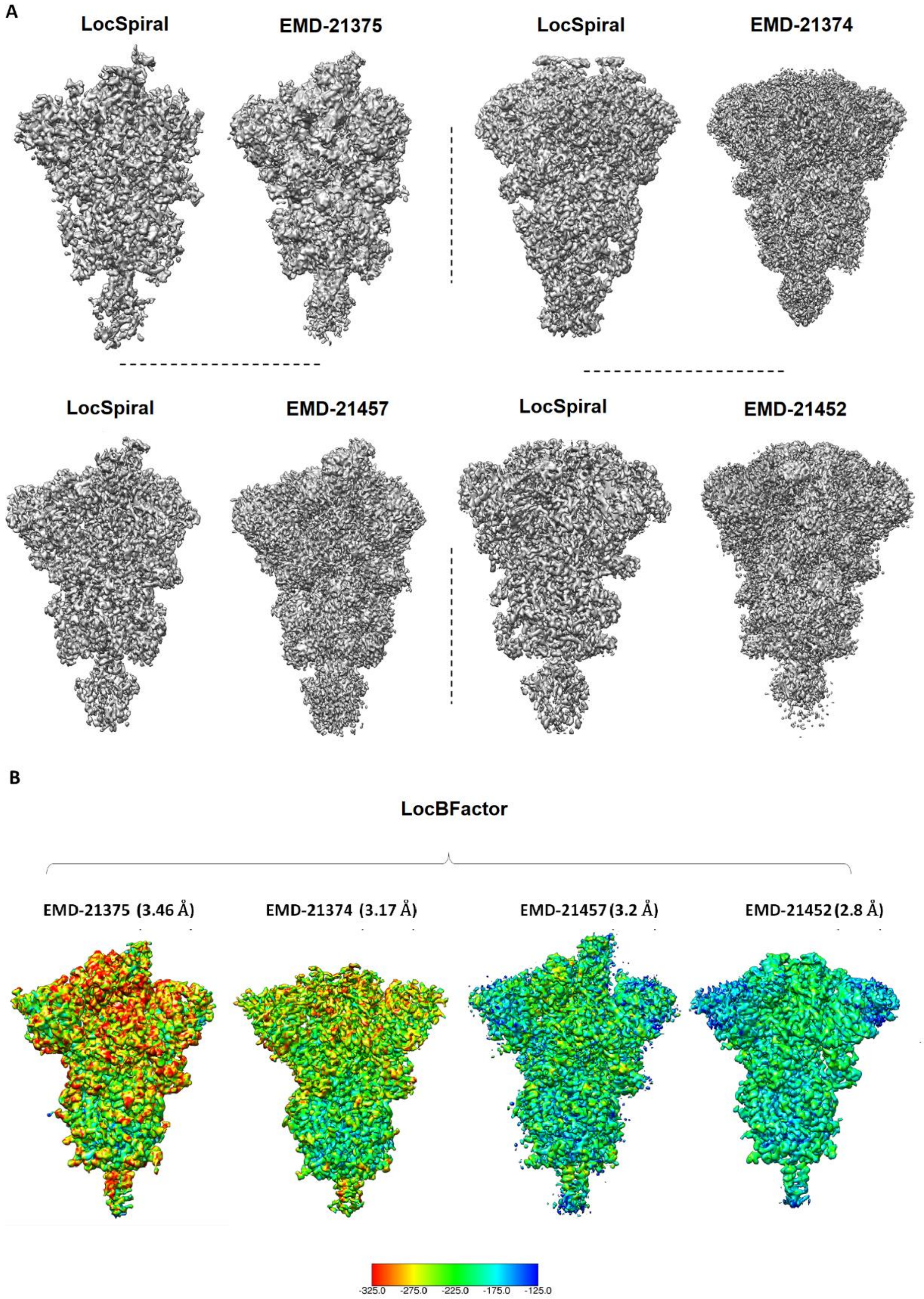

Supplementary Figure 6 Results obtained by LocSpiral, LocBFactor for SARS-CoV-2 samples. A) Maps obtained by LocSpiral approach (left) compared with maps as deposited in EMDB with accessing codes (EMD-21375, EMD-21374, EMD-21457, EMD-21452). B) Obtained B-factor maps to be used for sharpening (slope of the local Guinier plot multiplied by 4) by LocBFactor approach for EMD-21375, EMD-21374, EMD-21457, EMD-21452.

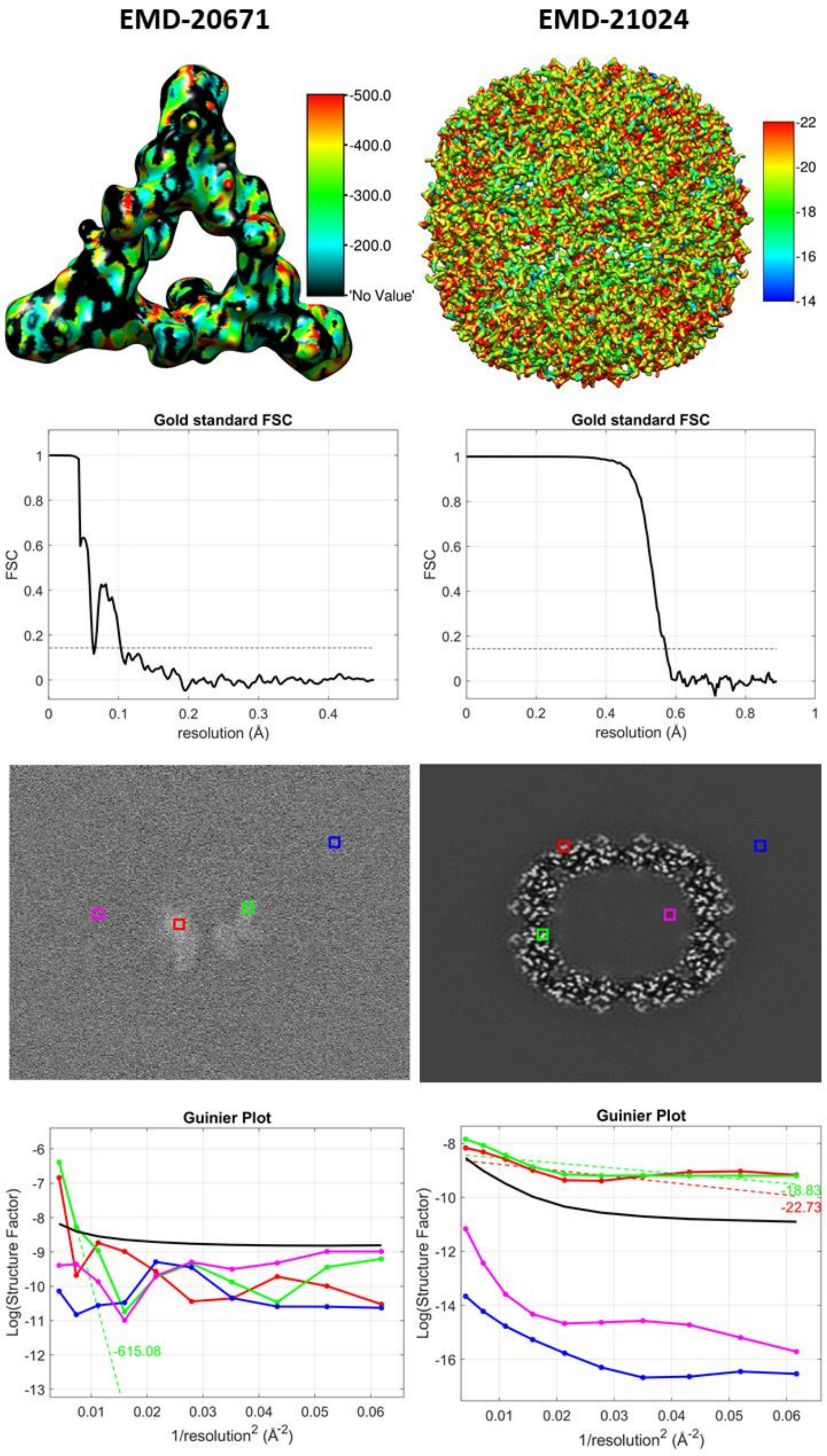

Supplementary Figure 7 Obtained B-factor maps (slope of the local Guinier plot) by LocBFactor approach for EMD-20671 and EMD-21024, corresponding FSC curves and Guinier plots of macromolecule and noise/background points indicated with coloured points shown in corresponding central slices.

Supplementary Table

		TRP channel (EMD-10418) (PDB 6t9n)	Apoferritin (EMD-9865) (PDB 6v21)	SARS-CoV-2 (EMD-21375) (PDB 6vsb)
**EMRINGER**	**EMRINGER LocSpiral**	2.31	8.63	2.31
	**EMRINGER Relion**	2.36	2.51	2.27
	Rotamer-ratio LocSpiral	0.70	0.97	0.70
	Rotamer-ratio Relion	0.72	0.67	0.73
	Max Z-score LocSpiral	7.93	48.83	9.47
	Max Z-score Relion	8.11	14.22	9.06
	Model Length LocSpiral	1184	3200	1683
	Model Length Relion	1184	3200	1598
MOLPROBITY	All-atom Clashscore LocSpiral	6.44	5.90	13.66
	All-atom Clashscore Relion	6.12	5.27	14.34
	Ramachandran Plot LocSpiral	Outliers:0.00% Allowed:4.38% Favored:95.62%	Outliers:0.00% Allowed:1.90% Favored:98.10%	Outliers:0.00% Allowed:8.36% Favored:91.46%
	Ramachandran Plot Relion	Outliers:0.00% Allowed:3.12% Favored:96.88%	Outliers:0.00% Allowed:2.33% Favored:97.67%	Outliers:0.00% Allowed:8.54% Favored:91.64%
	Rotamer Outliers LocSpiral	7.24 %	1.39 %	13.63 %
	Rotamer Outliers Relion	2.77 %	1.49 %	9.96 %
	Cbeta Deviations LocSpiral	0.00 %	0.00 %	0.00 %
	Cbeta Deviations Relion	0.00 %	0.00 %	0.00 %
	Peptide Plane LocSpiral	Cis-proline:0% Cis-general:0% Twisted Proline:0% Twisted-General: 0%	Cis-proline:25% Cis-general:0% Twisted Proline:0% Twisted-General:0%	Cis-proline:0.67% Cis-general:0% Twisted Proline:0.67% Twisted-General:0.03%
	Peptide Plane Relion	Cis-proline:0% Cis-general:0% Twisted Proline:0% Twisted-General: 0%	Cis-proline:25% Cis-general:0% Twisted Proline:0% Twisted- General:0%	Cis-proline:0% Cis-general:0% Twisted Proline:0% Twisted-General: 0%

Supplementary Table 1 EMRINGER and Molprobity modeling scores obtained between sharpened maps by Relion postprocessing and LocSpiral, and corresponding atomic models after refining the structure against corresponding maps by Phenix real_space_refine approach using 5 refining iterations

Supplementary References

1 Afonine, P. V. *et al.* New tools for the analysis and validation of cryo-EM maps and atomic models. *Acta Crystallogr D Struct Biol* **74**, 814-840, doi:10.1107/S2059798318009324 (2018).

2 Barad, B. A. *et al.* EMRinger: side chain-directed model and map validation for 3D cryo-electron microscopy. *Nat Methods* **12**, 943-946, doi:10.1038/nmeth.3541 (2015).

3 Wang, Q. *et al.* Lipid Interactions of a Ciliary Membrane TRP Channel: Simulation and Structural Studies of Polycystin-2. *Structure* **28**, 169-184 e165, doi:10.1016/j.str.2019.11.005 (2020).

4 Fernandez, J. J., Luque, D., Caston, J. R. & Carrascosa, J. L. Sharpening high resolution information in single particle electron cryomicroscopy. *J Struct Biol* **164**, 170-175, doi:10.1016/j.jsb.2008.05.010 S1047-8477(08)00144-5 [pii] (2008).

5 Kimanius, D., Forsberg, B. O., Scheres, S. H. & Lindahl, E. Accelerated cryo-EM structure determination with parallelisation using GPUs in RELION-2. *eLife* **5**, doi:10.7554/eLife.18722 (2016).

6 Davis, J. H. *et al.* Modular Assembly of the Bacterial Large Ribosomal Subunit. *Cell* **167**, 1610-1622 e1615, doi:10.1016/j.cell.2016.11.020 (2016).
